# Characterization of Acidic Tea Polysaccharides from Yellow Leaves of Wuyi Rock Tea and Their Hypoglycemic Activity via Intestinal Flora Regulation in Rats

**DOI:** 10.3390/foods11040617

**Published:** 2022-02-21

**Authors:** Zhong Wu, Wenzhi Zeng, Xun Zhang, Jiangfan Yang

**Affiliations:** 1 College of Horticulture, Fujian Agriculture and Forestry University, Provincial Key Laboratory of Tea Science in Universities, Fuzhou 350002, China; wzrenwz@163.com; 2 College of Pharmacy, Fujian University of Traditional Chinese Medicine, Fuzhou 350122, China; xunzhang716@163.com; 3 College of Agriculture, Fujian Agriculture and Forestry University, Fuzhou 350002, China; fafuzwz@163.com

**Keywords:** acidic tea polysaccharides, structure characterization, hyperglycemia, intestinal flora

## Abstract

A bioactive acidic tea polysaccharide from yellow leaves of Wuyi rock tea was successively prepared via DEAE-52 and Superdex-200 columns. Nuclear magnetic resonance (NMR) analysis showed that the main glycosidic bonds were composed of α-l-Araf-(1→, →5)-α-l-Araf-(1→, →4)-α-d-Glcp-(1→, Arap-(1→, →6)-α-d-Glcp-(1→, →2,4)-α-l-Rhap-(1→, →3,4)-α-d-Glcp-(1→, →4)-α-d-GalAp-(1→, →4)-α-d-GalAp-(1→, α-d-Galp-(1→, →6)-β-d-Galp-(1→ and →4)-β-d-Galp-(1→. The molecular weight was 3.9285 × 104 Da. The hypoglycemic effect of acidic tea polysaccharides on streptozotocin-induced type 2 diabetes mellitus rats was evaluated through histopathology and biochemistry analysis. The acidic tea polysaccharide could improve plasma and liver lipid metabolism. Moreover, 16S rRNA gene sequencing revealed that the composition of the intestinal flora changed drastically after treatment, namely, blooms of *Bifidobacterium*, *Blautia*, *Dorea*, and *Oscillospira*, and a strong reduction in *Desulfovibrio* and *Lactobacillus*. The above results illustrated that tea polysaccharides might serve as an effective ingredient to ameliorate glucose metabolism disorders and intestinal flora in hyperglycemic rats.

## 1. Introduction

Tea polysaccharide (TPS) is the main bioactive component of tea, especially in low-grade tea, with a content from 0.8% to 1.5% [1,2,3,4,5]. Many animal models and clinical studies have shown that tea polysaccharides are beneficial to human health, with antioxidant, antidiabetic, anti-inflammatory, and antitumor activity [1,6,7,8,9].

Tea polysaccharide is one of the most important bioactive components in tea and has received increasing levels of attention because of its non-toxicities. The hypoglycemic activity of tea polysaccharides is the most important and researched function. Numerous studies have shown that the hypoglycemic mechanism of tea polysaccharides is mainly achieved by regulating the activity of key enzymes in glucose metabolism and protecting pancreatic islet cells to promote insulin secretion [10]. Another prominent function of tea polysaccharides is antioxidant activity [1,3]. Antioxidant therapy to reverse the damage to tissue caused by oxidative stress has become a very effective strategy to prevent diabetes and its complications. Tea polysaccharides are now also believed to be able to be fermented by micro-organisms in the human intestine to produce products such as short-chain fatty acids (SCFAs), and these fermentation products interact with the intestinal flora to change the structure of the intestinal flora and affect the intestinal microecology [11], thus changing the course of diseases such as diabetes.

The structure of polysaccharides determines their biological activity [12]. The structure of tea polysaccharides can be divided into primary, secondary, tertiary, and quaternary structures, of which, secondary, tertiary, and quaternary structures are collectively referred to as advanced structures. The advanced structure is based on the primary structure [13,14]. The primary structure mainly includes the composition and arrangement order of the sugar groups, the connection of adjacent sugar groups, the anomeric carbon configuration, the presence or absence of branches in the sugar chain, and the position and length of the branch [7,15]. Compared with proteins and nucleic acids, the primary structure of polysaccharides is very complicated and is also the focus and difficulty of current research.

Takeo found that the polysaccharide extracted from tea with hypoglycemic activity was galactoglucan [15]. The tea polysaccharide isolated by Chen et al. with hypoglycemic effects was an acid ternary glycoconjugate containing protein and nucleic acid [16]. Wang et al. isolated protein-removing polysaccharides and demonstrated that they had a good stimulating effect on glucokinase [17]. Lu et al. found that acidic tea polysaccharide from Huangshan Maofeng could improve the activity of antioxidant enzymes and reduce the formation of peroxides in the liver of mice, thus achieving a hepatoprotective effect [18]. The combined literature shows that acidic tea polysaccharides have a wide range of biological activity. However, there are still only a few studies on the structure–activity relationship of tea polysaccharides.

Wuyi rock tea is one of the six major teas in China (black tea, green tea, oolong tea, yellow tea, white tea, and dark tea) commonly known as “oolong tea”. Wuyi rock tea was originally cultivated on Wuyi Mountain, located in the northern part of Fujian Province in southeastern China. Partly due to the unique climate and edatope of Wuyi Mountain, Wuyi rock tea is recognized as one of the most prestigious oolong teas due to its special savor and long-lasting fragrance, called the “rock bone floral fragrance” [19,20,21].

Wuyi rock tea is a semifermented tea with production methods ranging between green tea and black tea. The primary processing technology of Wuyi rock tea is: 1. picking, 2. withering, 3. fine manipulation of green tea leaves, 4. water removal, 5. rolling, and 6. drying [22,23,24,25,26]. The refining process technique of Wuyi rock tea is: 1. pick out the big stems and yellow leaves, 2. screen, 3. pick out the small stems and yellow leaves (relatively coarse and old leaves), 4. take the residue in its entirety, and then 5. use low-temperature long baking [22,23,24,25,26].

According to the estimate, byproducts such as yellow leaves and other inclusions account for approximately 20% to 30% of the total amount of crude tea. Yellow leaves are produced because the leaves have a thick layer of wax, and the pigment is not oxidized easily during processing and finally turns yellow. For leaves without wax protection, the color darkens during processing. Due to the poor appearance and inclusion ratio and the poor taste and aroma, yellow leaves are generally sold in low-price batches or used on tea trees as fertilizer, which has a low utilization rate.

The content of the main pharmacological components (such as caffeine, tea polyphenols, etc.) in the tea decreases with the degree of coarseness, while the content of tea polysaccharides is the opposite. The coarser the tea leaves, the higher the tea polysaccharide content. Therefore, we suggest that yellow leaves of Wuyi rock tea could be treated as a potential resource for the development of polysaccharide antioxidants and hypoglycemic products [1].

## 2. Materials and Methods

### 2.1. Extraction, Purification, and Identification of Purified Polysaccharide (CY)

The coarse tea of Wuyi rock tea (yellow leaves) was crushed into powder, and then 80% ethanol was added to remove fat-soluble ingredients. The crude polysaccharide was extracted with distilled water (1:30 solid/liquid ratio) and ultrasound-assisted extraction at 60 °C and 50 kHz for 2 h. The water extract was centrifuged (3000 rpm, 20 min, 20 °C) to collect the supernatant and then precipitated by the addition of 85% ethanol (4 °C, 24 h). The precipitate was collected through centrifugation (10,000 rpm, 20 min, 20 °C) and then freeze-dried to obtain the crude polysaccharide.

Crude polysaccharide (1 g) was dissolved with distilled water, heated, shaken well, and centrifuged at 12,000 rpm. The supernatant was added to a DEAE-52 cellulose chromatography column and then eluted at a 15 mL/min flow rate with distilled water and NaCl solution (0.2, 0.5, 2.0 mol/L sequentially). Eluent (4 mL) was collected in each tube. A completely separated fraction was gathered by measuring eluent absorbance at 490 nm according to the phenol–sulfuric acid method [27]. The fractions were dialyzed (3500 Da molecular weight cutoff (MWCO)) and lyophilized. Then, the fraction solution (100 mg/3 mL) was centrifuged at 12,000 rpm for 10 min. The supernatant was further purified using distilled water at a flow rate of 0.5 mL/min through Superdex-200 (GE Healthcare Bio-Sciences Corp., Marlborough, MA, USA) with refractive index detector (Shodex RI-502; Shoko Co., Ltd., Kanagawa, Japan). After lyophilization, the purified polysaccharide named CY was obtained. The purity of CY measured was 91.3% using high-performance liquidchromatography (HPLC).

### 2.2. Molecular Weights and Monosaccharide Composition Measurement

The average molecular weights of CY were measured using a Shimadzu high-performance gel permeation chromatography (HPGPC) system equipped with a BRT105-104-102 (8 × 300) column and a Shimadzu RI-502 refractive index detector. The sample size was 20 μL, and 0.05 mol/L NaCl solution was used as the mobile phase at a flow rate of 0.6 mL/min. The column temperature was controlled at 40 °C during the operation. The standard curve was established with a dextran standard.

The monosaccharide composition of CY was determined by an ion spectrometer (ICS5000; Thermo Fisher Technology Co., Ltd., Waltham, MA, USA) with a DionexCarbopac^TM^PA20 chromatographic column (3 × 150) and an electrochemical detector. The sample size was 5 µL, and H_2_O, 250 mmol/L NaOH, 50 mmol/L NaOH, and 500 mmol/L NaOAC were used as the mobile phases at a flow rate of 0.3 mL/min. The column temperature was controlled at 30 °C during operation. The monosaccharide standards include fucose, rhamnose, arabinose, glucosamine hydrochloride, galactose, glucose, N-acetyl-d-glucosamine, xylose, mannose, fructose, ribose, galacturonic acid, and glucuronic acid.

### 2.3. FTIR Spectroscopy Analysis

The CY sample was measured in the region of 4000–400 cm^−1^ by a Fourier transform infrared spectrometer (FTIR). The mixture (2 mg CY mixed with 200 mg KBr) was pressed into transparent sections by a cylindrical mold for FTIR analysis.

### 2.4. Glycosidic Linkage Type Analysis

Methylation and gas chromatography/mass spectrometry (GC/MS) analysis were performed according to the method described by Shi et al. [28]. Briefly, 3 mg of dried CY was dissolved in 1 mL dried dimethyl sulfoxide (DMSO). Then, 20 mg of NaOH powder was added, and the mixture was dissolved under ultrasonic action. The methylation reaction was maintained for 1 h after the slow addition of CH_3_I (1.0 mL) to the system. Then, 2.0 mL of ultrapure water was added to terminate the reaction. After the extraction of the methylated polysaccharide, 1 mL of 2 mol/L trifluoroacetic acid was added and hydrolyzed for 90 min. After drying, 2 mL of distilled water and 60 mg of sodium bromide were added, stirred at room temperature for 8 h, neutralized with glacial acetic acid, and dried at 101 °C. Finally, 1.0 mL of acetic anhydride was added, acetylated at 100 °C for 1 h, and cooled. The product was extracted with 3 mL of methylbenzene, depressurized, and dried three times to remove acetic anhydride. The product was transferred to 3 mL dichloromethane, a small amount of distilled water was added, and it was shaken well to remove the upper aqueous solution, repeated four times. The dichloromethane layer was dried by anhydrous sodium sulfate in a constant volume to 10 mL. The concentrated methylated alditol acetate derivatives were filtered through a 0.22 μm membrane for GC/MS analysis (GCMS-QP2010; Shimadzu Instruments Co., Ltd., Kyoto, Japan).

### 2.5. Nuclear Magnetic Resonance (NMR) Spectrometer Analysis

CY (50 mg) was accurately weighed and dissolved in D_2_O to exchange hydrogen atoms for deuterium and then freeze-dried, repeated threetimes to obtain the sample. ^1^H and ^13^C NMR spectra of the CY sample were determined using a Bruker DRX-600 NMR spectrometer (Bruker BioSpin GmbH, Rheinstetten, Germany). MestReNova (Mwstrelab Research, Santiago de Compostela, Spain) software was used to process and analyze the NMR spectra results.

### 2.6. Ethics Statement

The animal experiment was approved by the Institutional Animal Care and Use Committee of Fujian University of Traditional Chinese Medicine (approval no. 2018070), and all guidelines for the care and use of animals were followed.

### 2.7. Animal Preparation and Experiment Design

Sixty male Wistar rats (6 weeks old, 200 ± 20 g) were provided by Shanghai SLAC Laboratory Animal Co., Ltd. (Shanghai, China). All experimental rats were raised under stable conditions (12 h daylight cycle, 55 ± 10% humidity, and 22 ± 2 °C) with free food and water. After 1 week of adaptive feeding, animals were randomly distributed into 6 groups. Normal control (NC) group rats were provided a chow diet, while the others were fed a high-fat diet (2.5% cholesterol, 1.0% sodium cholate, 20.0% sugar, 10.0% lard, and 66.5% commercial standard pellet diet). After 4 weeks of dietary manipulation, all rats were fasted for 12 h. Rats fed a high-fat diet were administered streptozotocin (STZ, 30 mg/kg body weight) intraperitoneally (i.p.) to induce type 2 diabetes mellitus (T2DM), and those fed a basal diet received an equivalent volume of saline. The rats were considered diabetic when the fasting blood glucose (FBG) level exceeded 16.7 mmol/L. The diabetic rats were distributed to the groups treated with 0 mg/kg CY (abbreviated as DC), 200 mg/kg CY (abbreviated as LT), 400 mg/kg CY (abbreviated as MT), 800 mg/kg CY (abbreviated as HT), and 200 mg/kg metformin (abbreviated as ME).

CY and metformin were diluted with distilled water to the desired concentration prior to gavage. The blood glucose value was measured via blood obtained from rat tail veins.

### 2.8. Sample Collection and Biochemical Index Analysis

During the experiment, the mental state and activity of the animals were observed daily, and the food intake and body weight were quantitatively determined weekly.

After fasting for 12 h, the rats were anesthetized using pentobarbital sodium to gather blood samples at the end of the observation period. The blood samples were centrifuged (3500 rpm, 15 min, 4 °C) to obtain clear serum. The serum levels of TG, TC, HDL-C, LDL-C, AST, and ALT were measured by assay kits (Nanjing Jiancheng Bioengineering Institute, Nanjing, China) according to the manufacturer’s protocol.

### 2.9. Measurement of Fasting Glucose and Oral Glucose Tolerance Test (OGTT)

On days 10, 20, 30, and 40, after the rats were fasted without water for 6 h, the fasting blood glucose values of the rats were measured by tail vein blood sampling before and after drug administration. At the end of the observation period, all rats were selected for the oral glucose tolerance test (OGTT). After 12 h of fasting overnight, basal blood glucose was detected, followed by gavaging each rat with glucose solution (1.5 g/kg). Then, blood glucose was monitored via blood obtained from rat tail veins at the following time points: 0 min, 30 min, 60 min, and 120 min. The results of the OGTT are expressed as the area under the glucose curve (AUC) over 120 min.

### 2.10. Histopathological Examination

The rat livers and pancreatic tissue were weighed, and hematoxylin and eosin (HE) staining was performed for histological investigation. In detail, the samples were fixed with 4% paraformaldehyde, dehydrated with alcohol gradients, cleared with xylene, embedded in paraffin, and sectioned. After deparaffinization, the sections were subjected to hematoxylin and eosin staining and then observed under a high-magnification microscope.

### 2.11. 16S rRNA Gene Sequence Analysis of Gut Microbiota in Cecum

Cecum contents were frozen in liquid nitrogen immediately after harvest and stored at −80 °C until sequencing. The sequencing service was conducted by Shanghai Personal Biotechnology Co., Ltd. (Shanghai, China). Total DNA from the cecal contents was extracted with Fast DNA SPIN extraction kits (MP Biomedicals, Santa Ana, CA, USA) following the instruction manual. The bacterial 16S rRNA gene V3-4 region was amplified by polymerase chain reaction (PCR) using the forward primer (5′-ACTCCTACGGGAGGCAGCA-3′) and the reverse primer (5′-GGACTACHVGGGTWTCTAAT-3′). PCR amplicons were purified with VAHTSTM DNA Clean Beads (Vazyme, Nanjing, CN) and quantified individually using the PicoGreen dsDNA Assay Kit (Invitrogen, Carlsbad, CA, USA). Then, amplicons were pooled in equal amounts, and paired-end 2 × 300 bp sequencing was performed on the Illumina MiSeq platform, as described by Yang et al. [29]. The sequencing data and predicted functional data were obtained by QIIME2 (2019.4) and PICRUSt2. The following statistics were performed by R software. α-Diversity evaluation was calculated by Chao1, observed species, Simpson, Shannon, Faith’s PD, Pielou’s evenness, and Good’s coverage index. β-Diversity evaluation was assessed with UniFrac distance-based principal coordinate analysis (PCoA) and nonmetric multidimensional scaling (NMDS). Linear discriminant analysis with effect size (LEfSe), heatmaps, and random forest classifier was applied to identify specific taxa of microbes among groups using the default parameters [30,31]. Zi (within-module connectivity) and Pi (among-module connectivity) score values were performed to search the keystone species. The predicted genes and their functions were aligned to the Kyoto Encyclopedia of Genes and Genomes (KEGG) database, and differences among groups were compared through the PICRUSt2 software [32,33].

### 2.12. Statistical Analysis

All the data in this paper are expressed as the mean±standard deviation (SD) from three independent experiments conducted in parallel, and Student’s test was used for the statisticalanalysis. Statistical data showed a significant difference when the *p* value was less than 0.05 and a highly significant difference when the *p* value was less than 0.01. Microbiological analysis was performed using the statistical software R (3.4.1, Robert Gentleman and Ross Ihaka, Auckland University, Auckland, New Zealand).

## 3. Results

### 3.1. Structural Characterization of CY

#### 3.1.1. Preparation, Molecular Weight, and Monosaccharide Composition of CY

**Figure 1:**
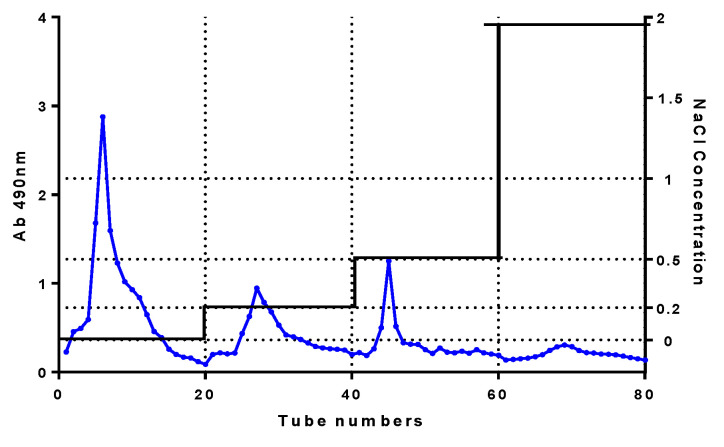
Elution diagram of DEAE-52.

The four peaks from left to right correspond to the solvents of water, 0.2 mol/L NaCl, 0.5 mol/L NaCl, and 2.0 mol/L NaCl, respectively;the eluted components were neutral polysaccharide (solvent was water) and acidic polysaccharide; and the yields of each component were 2%, 9.4%, 2.9%, and 0.6%, respectively (Figure 1). The acidic tea polysaccharide isolated by 0.2 mol/L NaCl was further purified by subsequent experiments and named CY.

**Figure 2:**
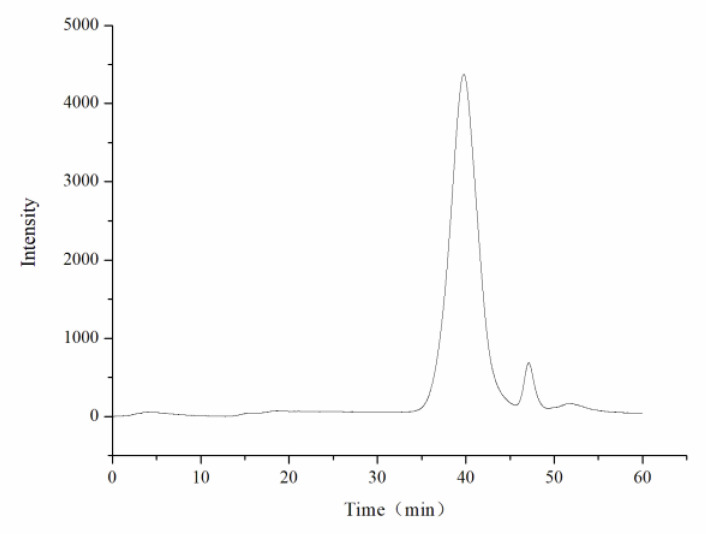
HPGPC chromatogram of the polysaccharide from yellow leaves of Wuyi rock tea (CY).

**Figure 3:**
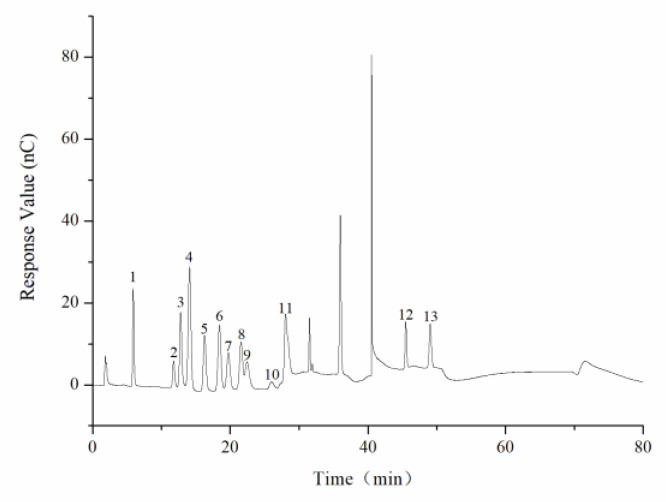
Ion chromatogram of mixed monosaccharide standards. 1. Fuc, 2. Rha, 3. Ara, 4. GlcN, 5. Gal, 6. Glc, 7. GlcNAc, 8. Xyl, 9. Man, 10. Fru, 11. Rib, 12. GalA, 13. GlcA.

**Figure 4:**
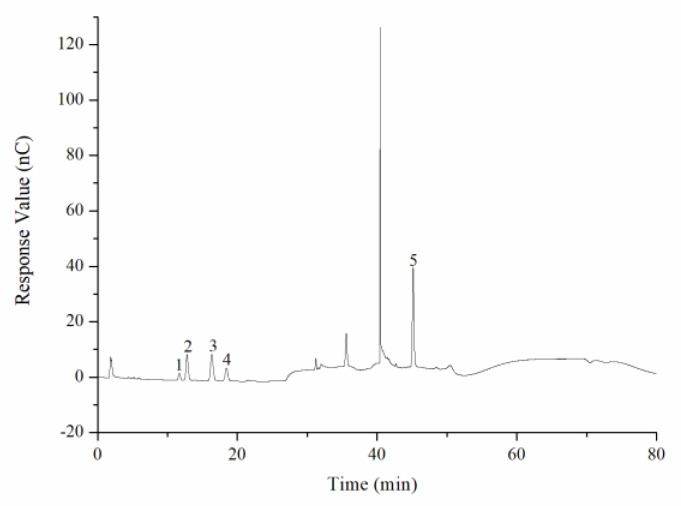
Ion chromatogram of component monosaccharides released from CY. 1. Rha, 2. Ara, 3. Gal, 4. Glc, 5. GalA.

The average molecular weight was determined by HPGPC. A series of dextran standard references was used for calibration curve establishment. The results showed that the average molecular weight of CY was 39,285 Da (Figure 2, Table 1). The monosaccharide composition was determined by ion spectrometry. CY consisted of arabinose (Arab), galactose (Gal), rhamnose (Rha), and glucose (Glc) in a mole ratio of 0.124:0.143:0.086:0.02, and the galacturonic acid (GalA) content of CY was 62.7% (Figure 3 and Figure 4, Table A1).

#### 3.1.2. FTIR Analysis of CY

**Figure 5:**
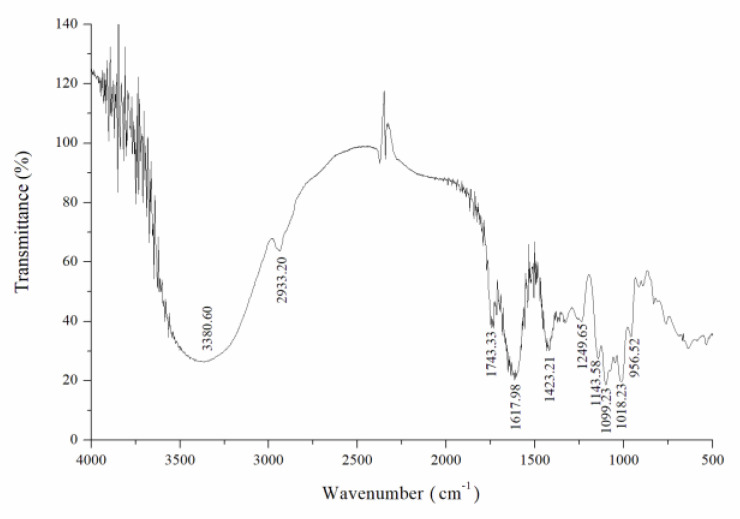
FTIR spectra of tea polysaccharide (CY).

The infrared spectra of CY are shown in Figure 5. The absorption band at 3600–3200 cm^−1^ was regarded as the stretching vibration absorption peak of -OH. The absorption peak in this area was the characteristic peak of saccharides. The stretching vibration absorption peak of O-H was centered at 3380 cm^−1^ and was a characteristic peak of saccharides. The weak absorption peak at 2933 cm^−1^ was attributed to the C-H stretching vibration. In addition, there was a strong absorption peak at 1743 cm^−1^ ascribed to the C=O stretching vibration, and the absorption peak at 1616 cm^−1^ reflected the C=O asymmetric stretching vibration, indicating that the component contains uronic acid, which was another characteristic peak of saccharides. The absorption peak at 1423 cm^−1^ showed variable angle vibration of C-H. The absorption peak caused by C-O stretching vibrations at 1200–1000 cm^−1^ was mainly caused by the vibrations of C-O-H and C-O-C saccharide ring, which were specifically manifested at 1143 cm^−1^, 1099 cm^−1^, and 1018 cm^−1^, indicating the presence of pyranose rings in this component. Compared with the infrared spectra of crude tea polysaccharide from yellow leaves of Wuyi rock tea in our previously published paper [34], the absorption peaks of the amide I band and amide II band disappeared in the purified CY, and the disappearance of these two protein absorption characteristic peaks indicated that the residual proteins had been removed.

#### 3.1.3. Methylation Analysis of CY

To further characterize the structure of CY, CY was completely methylated, acid-hydrolyzed, and acetylated: the unmethylated part was the connection site between the glycosyl groups. www.ccrc.uga.edu (accessed on 22 April 2020), the related literature [35,36,37,38], and the Mass Spectrometry Database of the Shanghai Institute of Organic Chemistry were used to analyze the data.

**Figure 6:**
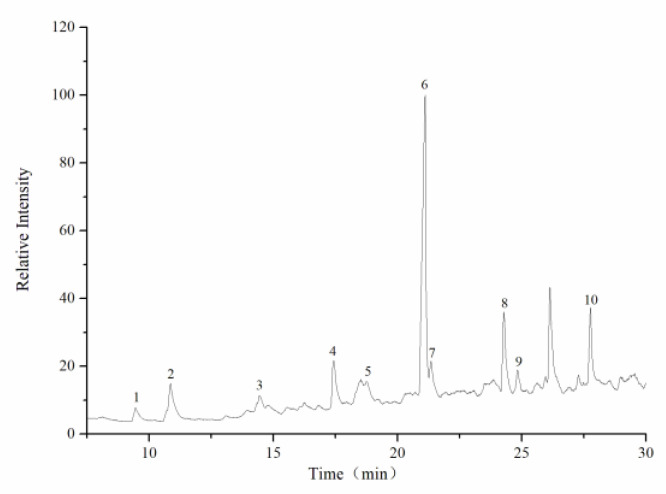
Type of polysaccharide linkage. 1. Araf-(1→ 2. Arap-(1→ 3. →5)-Araf-(1→ 4. Galp-(1→ 5. →2,4)-Rhap-(1→ 6. →4)-Galp-(1→ 7. →4)-Glcp-(1→ 8. →6)-Galp-(1→ 9. →3,4)-Glcp-(1→ 10. →4,6)-Glcp-(1→.

Figure 6 and Table A2 shows the CY methylation analysis results. As shown in Figure 6 and Table A2, the linkage patterns and corresponding molar percentages of CY are summarized, indicating that CY consisted mainly of 10 linkage types, and the ratio of each linkage type is different. This tea polysaccharide contains at least three branch points: →2,4)-Rhap-(1→, →3,4)-Glcp-(1→, →4,6)-Glcp-(1→. Among these three branch points, →4)-Galp-(1→ accounts for a large proportion. →4)-Galp-(1→ was presumed to constitute the main chain part of CY; →5)-Araf-(1→ and →6)-Galp-(1→ were presumed to be the branched part of CY. The content of Araf-(1→, Arap-(1→, and Galp-(1→ was relatively low, and we speculated that the three mainly exist at the end of the branch chain and the nonreducing end of the main chain. In summary, the main part of CY was a 1,4-linked main chain, and 1,6-, 1,5-linked residues were branched-chain polymeric structures. According to the analysis, this result was consistent with the composition of monosaccharides determined by ion chromatography analysis.

#### 3.1.4. NMR Analysis

NMR spectroscopy is widely used to analyze the structure of complex polysaccharides. The residues of polysaccharides could be determined by assessing the main anomeric proton signals of the ^1^H- and ^13^C-NMR spectra. The signals of the proton in ^1^H NMR spectra were mainly concentrated at δ3.0~5.5 ppm, which was a specific feature of polysaccharides. The signals in the range of δ3.2–4.0 ppm were assigned to protons in the sugar ring. The signal peaks of the main terminal group matrix proton peaks are concentrated in the range of 4.3~5.5 ppm.

In the ^13^C NMR (126 MHz, D_2_O) spectra, nuclear magnetic carbon spectrum signals were mainly concentrated between 20 and 180 ppm. By observing the carbon spectra, the main anomeric carbon signal peaks could be seen to mainly range betweenabout δ93 and 110, which existed at δ108.85, 101.76, and 100.87. The main signal peaks are distributed in the range of 60~85 ppm and occurred at δ83.71, 82.73, 80.42, 79.10, 77.96, 74.72, 72.96, 71.94, 69.84, 69.39, 68.18, and 62.55 ppm. The signal peaks at 176.66 and 172.19 ppm suggested the existence of carbonyl carbon. The signal peaks at δ54.28 ppm revealed the existence of methyl ester carbon. The signal at δ18.0 ppm was attributed to the C-6 of rhamnose residues. In addition, δ21.2 ppm implied the existence of uronic acid. By the Dept135 spectral analysis, the signals of δ61.86 ppm and 62.09 ppm were found to be inverted peaks, indicating a chemical shift of C-6.

**Figure A1:**
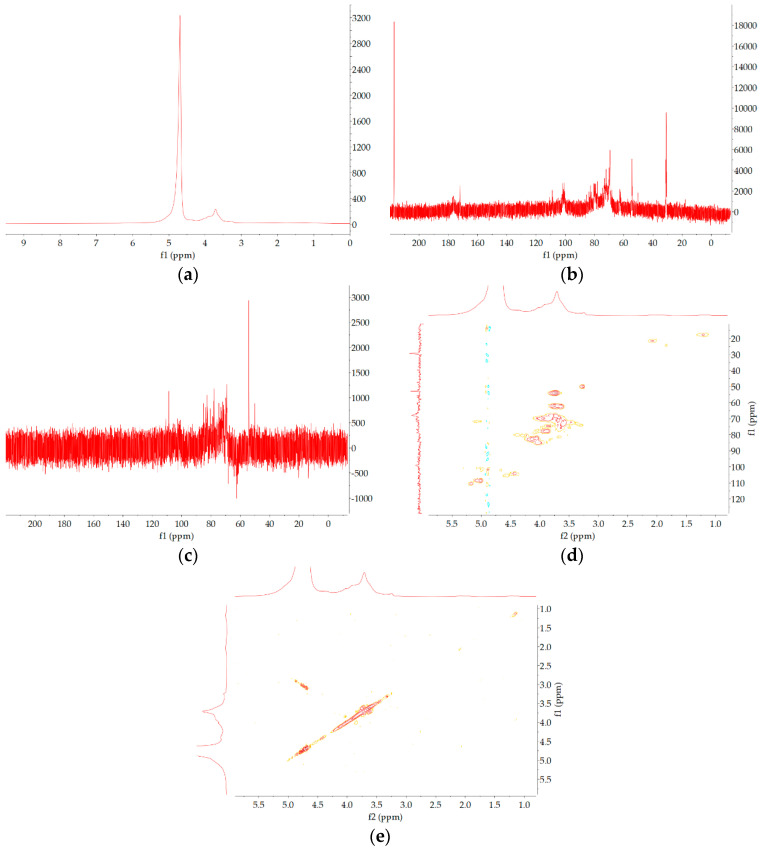
^1^H- (**a**) and ^13^C-NMR (**b**), DEPT135 (**c**), HSQC (**d**), and COSY (**e**) spectra of CY in D_2_O.

Through the HSQC spectra, the anomeric carbon signal was observed to be δ 108.85, and the corresponding anomeric hydrogen signal in the HSQC spectrum was δ5.04. Through HH-COSY, the signal of H1-2 was 5.04/4.07, the signal of H2-3 was 4.07/3.96, the H3-4 signal was 3.96/4.16, and the H4-5 signal was 4.16/3.75. We could infer that H1, H2, H3, H4, and H5 were δ5.04, 4.07, 3.96, 4.16, and 3.75, respectively. The corresponding C1-5 values were 108.85, 82.52, 78.12, 83.71, and 68.18, respectively. Therefore, the signal should be attributed to →5)-α-L-Araf- (1→. Similar rules were used to assign glycosidic bonds according to references [39,40,41,42] as follows (Figure A1 and Table A3).

### 3.2. Effect of CY on T2DM Rats

#### 3.2.1. General Observation of Rats

During the experiment, the rats in the NC group were in good condition, with slow and continuous weight gain, good mental state, free movement, shiny hair, moderate diet, drinking water, urine volume, and normal stool. In the model rats, the body weight increased rapidly during high-fat and high-sugar feeding. After intraperitoneal injection of STZ, there was significant weight loss; decreased activity; hair loss and lack of luster; a significant increase in diet, water intake, and urine output; and thin and unformed feces.

After drug intervention, compared with the DC group, the rats in the tea polysaccharide intervention groups (LT, MT, HT) and the metformin intervention group (ME) had smoother hair and reduced shedding, significantly improved mental state, increased mobility, and no further weight loss. There was a small cut in diet, water, and urine, and stool was more formed. During the entire experiment, two rats were not modeled with STZ, two died in the DC group, and one died in the low-dose tea polysaccharide group (LT), which may be due to long-term hyperglycemia leading to ketoacidosis.

#### 3.2.2. Effect of CY on Fasting Blood Glucose in Diabetic Rats

The changes in fasting blood glucose values of diabetic rats before and after the administration of the drug are shown in Table A4. As seen from the table, the blood glucose values of rats in the NC group and the DC group fluctuated over a small range, and the blood glucose values were more stable in different periods. The blood glucose of the rats in the drug-treated group changed significantly, and the ME group showed the largest range of blood glucose changes, with a decrease of 41.72%. The rats in the high-, medium-, and low-dose groups of tea polysaccharide also had certain hypoglycemic effects, among which the MT group had the best hypoglycemic effect, and the difference between the blood glucose values of the diabetic rats after 40 days of tea polysaccharide intervention (MT group) and the DC group was significant (*p* < 0.05).

#### 3.2.3. Effect of CY on Glucose Tolerance in Diabetic Rats

Glucose tolerance was measured in rats at day 38 of administration. The results showed that the glucose values in the DC group were significantly higher than the glucose values in the NC group after 0, 30, 60, and 120 min of glucose loading, and the glucose values in the diabetic rats increased rapidly during the first 30 min and reached a peak, then decreased slowly and approached the starting value at 120 min. The blood glucose of rats in the ME group at this moment showed significant differences from that of rats in the DC group (*p* < 0.05).The area under the glucose curve (AUC) is often used to reflect the extent to which the body uses and clears glucose, and an increase in its value indicates a decrease in the body’s glucose tolerance. The DC group had the largest AUC, indicating the greatest decrease in glucose-tolerance capacity. The AUC in the DC group showed significant differences to those of rats in the ME, MT, and HT groups (*p* < 0.01) (Table A5).

#### 3.2.4. Biochemical Indices in Diabetic Rats

Serum alanine aminotransferase (ALT) and aspartate aminotransferase (AST) levels are closely related to liver cell damage, and liver fat accumulation is another important factor leading to liver cell damage. There is also a close relationship between serum ALT and AST levels and T2DM, which could predict the occurrence of T2DM. Serum AST and ALT levels in the DC group were significantly higher than those in the NC group. After tea polysaccharide intervention, the serum AST and ALT levels were apparently decreased. The triglyceride (TG) and LDL-C content in the DC group were significantly increased compared with the contents in the NC group (*p* < 0.01), demonstrating that the diabetic rats had developed a disorder of lipid metabolism. Compared with the DC group, the serum triglyceride level was significantly reduced after tea polysaccharide intervention (*p* < 0.05). The differences in the HDL-C and TC levels between the DC group and the tea polysaccharide intervention groups were not statistically significant (*p* > 0.05). The difference in LDL-C levels between the DC group and the HT group was statistically significant (*p* < 0.05) (Table A6).

#### 3.2.5. Pathological Tissue Section Observations

##### Effect of CY on Liver Cell Morphology in Diabetic Rats

**Figure 7:**
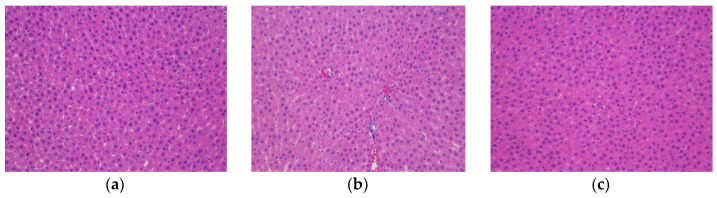

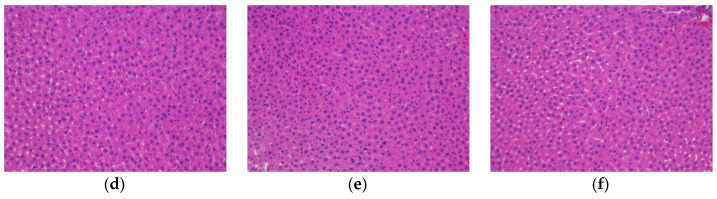
The effects of CY on liver cell morphology in diabetic rats (×200). (**a**) NC; (**b**) DC; (**c**) ME; (**d**) LT; (**e**) MT; (**f**) HT.

The results of HE staining showed that the liver tissue of the NC group was structurally intact, with regular cell arrangement, consistent size and radial distribution, rich cytoplasm of hepatocytes, no obvious degeneration ornecrosis, clear and centrally located nuclear structure, and no lymphocyte infiltration. In the DC group, there were obvious changes in liver structure and poor hepatocyte integrity; some cells were lysed, the structural outline was blurred, and there were reduced nuclei, swelling of hepatocytes, and inflammatory cell infiltration. The hepatocyte structure improved in each TPS treatment group compared to the DC group, with fewer lipid droplets and less inflammatory cell infiltration than in the DC group (Figure 7).

##### Effect of CY on Pancreatic Tissue Morphology in Diabetic Rats

**Figure 8:**
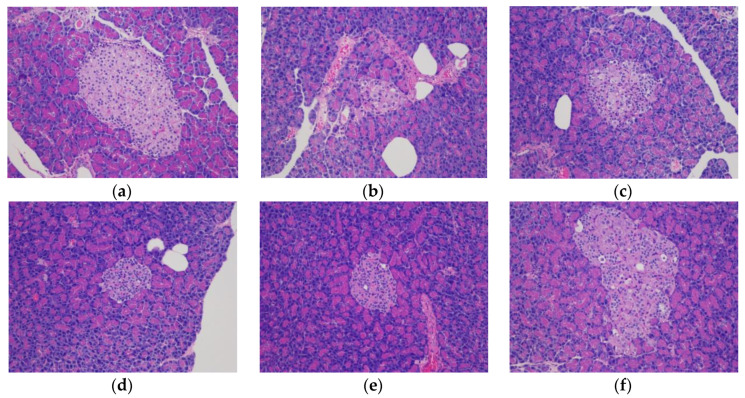
The effects of CY on pancreatic islet morphology in diabetic rats (×200). (**a**) NC; (**b**) DC; (**c**) ME; (**d**) LT; (**e**) MT; (**f**) HT.

Observation of HE staining of pancreatic tissue revealed that the pancreatic tissue of the NC group had no abnormal morphology, the marginal boundaries were clear and noninfiltrating, and the islet cells in the pancreatic islets were neat and orderly, with similar shapes and consistent sizes. In the DC group, the islet shape was irregular; the margins were unclear and infiltrated; the number of islet cells in the islet was small, disordered, unevenly distributed, and inconsistent in size; and some cells had vacuoles, deformed shape, and necrosis. Compared with the DC group, the tea polysaccharide treatment groups had less β-cell destruction, and all of the tea polysaccharide treatments improved the distribution, cell morphology, and staining granule distribution within the islets to different degrees. The number of stained granules of pancreatic β-cells increased and arranged into clusters, with a clearer cell edge structure and less vacuolization than the DC group (Figure 8).

### 3.3. Variations in Intestinal Flora in T2DM Rats after CY Treatment

#### 3.3.1. Species Annotation

**Figure A2:**
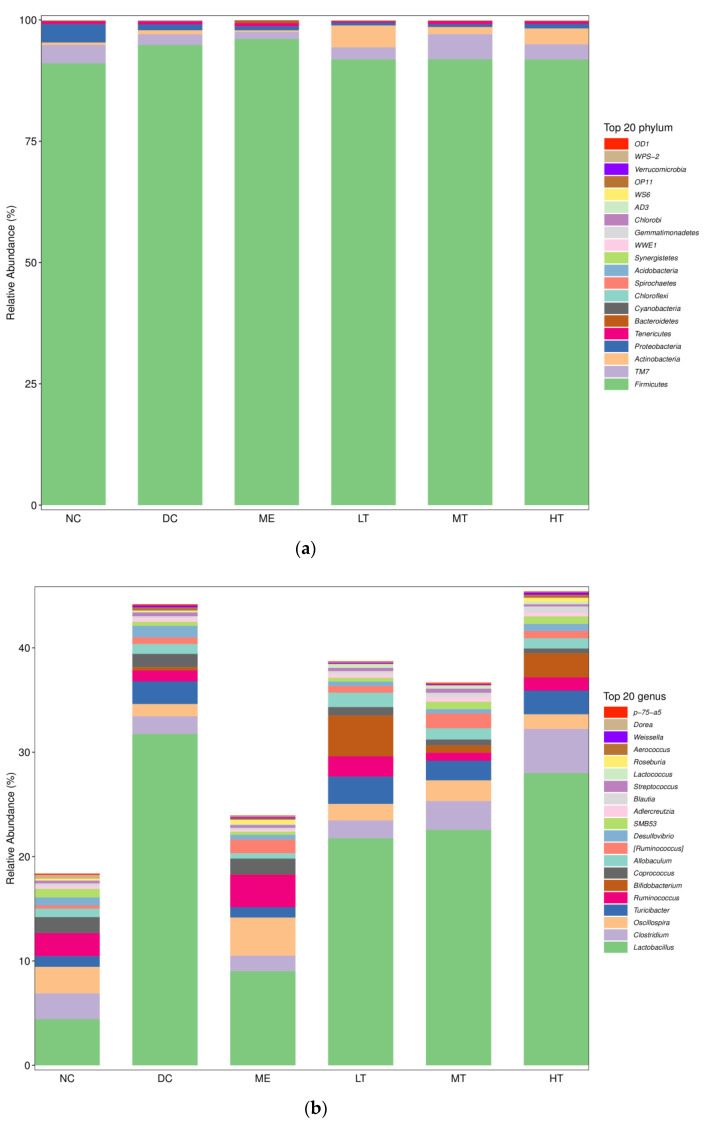
Phylum- and genus-level distributions of fecal microbiota. (**a**) Phylum-level distribution; (**b**) genus-level distribution.

According to species annotation, the statistical number of sequences of every group at the phylum and genus levels was calculated. The sequence constitution histogram of species is shown as follows. Accordingly, the top 20 species at the phylum level were selected, and the distribution histogram of the relative abundance of species was formed, as shown in Figure A2a. The top 20 species at the classification level of the genus were selected, and the distribution histogram of the relative abundance of species was formed as follows in Figure A2b.

As shown in Figure A2a, demonstrating the intestinal flora of the first 20 phyla of each group of rats, the abundance of Firmicutes had a significant advantage. Compared to the DC group, the TM7 and Actinobacteria abundance rebounded significantly (*p* < 0.01) in each dose of the tea polysaccharide intervention group. The regularity of the changes in the number of other phyla was not obvious, presenting the complexity of the growth of intestinal flora.

The samples of each group differed in as many as 248 species of flora at the genus level, and we further analyzed only the top 20 genera with the highest proportion of distribution. Compared to the NC group, after successful modeling, we found that the proportion of the top 20 genera in the DC group increased significantly, implying that the DC group became less diverse and uneven with decreased diversity. Among these genera, *Lactobacillus* belonging to the Firmicutes was significantly increased (*p* < 0.01), and the total amount reached approximately 30%; *Streptococcus*, *Turicibacter*, and *Desulfovibrio* were significantly increased (*p* < 0.01), while *Oscillospira*, *Ruminococcus*, *Clostridium*, *Blautia*, *Roseburia*, and *Dorea* significantly decreased (*p* < 0.01). Compared with the DC group, the proportion of the top 20 genera in the ME group decreased significantly after metformin intervention, indicating a significant improvement in the balance of species and proportions of the flora. Compared with the DC group, the proportions of *Bifidobacterium*, *Blautia*, *Dorea*, and *Oscillospira* increased significantly (*p* < 0.01), while the proportions of *Desulfovibrio* and *Lactobacillus* decreased significantly (*p* < 0.01) in the tea polysaccharide intervention groups.

#### 3.3.2. Alpha Diversity

Microbial diversity could be assessed within a community (alpha diversity) or between the collection of samples (beta diversity). Seven different metrics were calculated to assess the alpha diversity: “Chao1” estimates the species abundance; “Observed Species” estimates the number of unique operational taxonomic units (OTUs) found in each sample; “Simpson index” and “Shannon index” give the measurement of both species number (richness) and the distribution of the abundance (evenness); “Faith’s PD index” characterizes evolutionary-based diversity; “Pielou’s evenness index” estimates the uniformity; and “Good’s coverage index” estimates the coverage.

**Figure A3:**
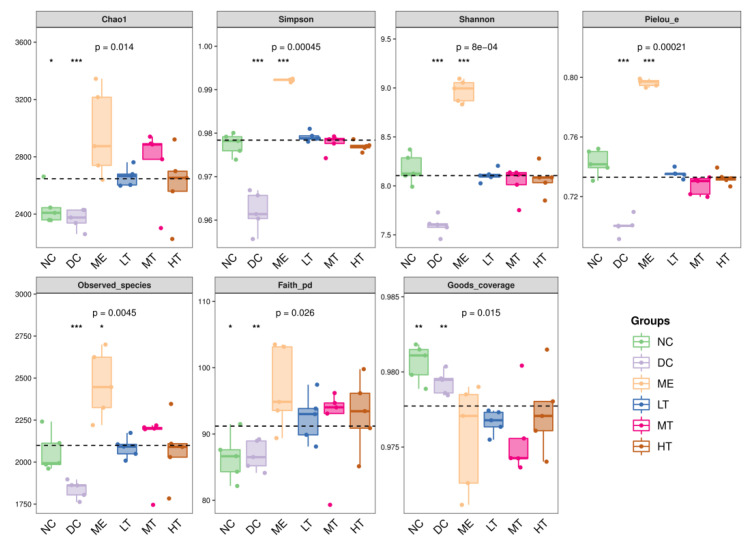
Grouped box plot of Alpha Diversity Index.Note: Each panel corresponds to an alpha diversity index, which is identified in the gray area at the top of the panel. The numbers under the diversity index label are the *p* values of the Kruskal–Wallis test. * represents a significant difference between the subgroup below it and the whole formed by the samples of all the remaining subgroups (*p* < 0.05); ** represents a highly significant difference (*p* < 0.01); *** represents a highly significant difference (*p* < 0.001).

Higher Chao1 and observed species indices indicate higher community richness. Higher Shannon’s index and Simpson’s index values indicate higher community diversity. A higher Pielou’s evenness index indicates more homogeneous communities. The higher Faith’s PD index is, the higher the degree of genetic diversity of the community. The higher Good’s coverage index is, the lower the proportion of undetected species in the sample. As seen from the box line plot, the diversity and richness of the flora decreased in Wistar rats after streptozotocin modeling (*p* < 0.05), and the alpha diversity index rebounded after metformin and tea polysaccharide intervention, indicating that the diversity and richness of the flora rebounded. The results were consistent with the previous analysis of species composition (Figure A3).

#### 3.3.3. Beta Diversity

##### Principal Coordinate Analysis (PCoA)

Beta diversity metrics assess the differences between microbial communities. They can be visualized with analyses such as principal coordinate analysis (PCoA) and non-measured multidimensional scaling (NMDS).

**Figure 9:**
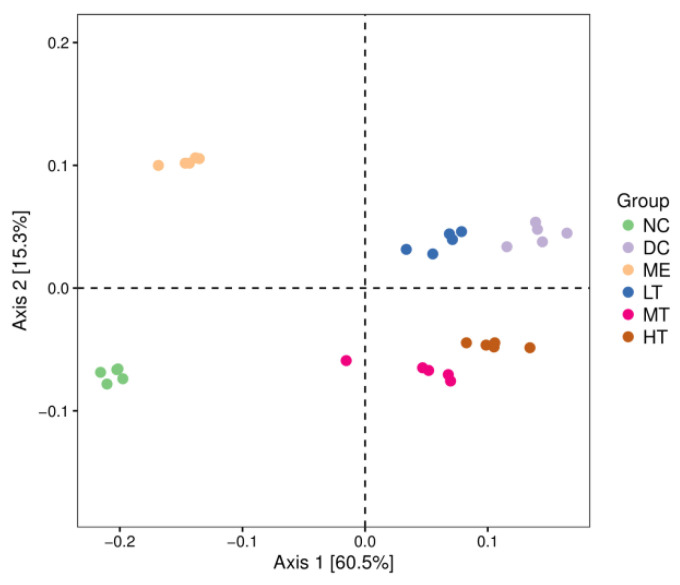
PCoA of weighted distance.

PCoA analysis showed the similarities and differences of the colonies in different environments. Each point in the graph represents a sample colony, and the distance between points represents the similarity between colonies; the smaller the distance is, the more similar the colonies are. The contributions of the first principal component (Axis 1) and the second principal component (Axis 2) of the model were 60.5% and 15.3%, respectively, based on the weighted principal coordinate analysis of the UniFrac distance. From the PCoA plot, the five samples within each group can be seen to have obvious aggregation phenomena, and the samples among the six groups are clearly discrete, which shows that the diversity of the flora among the groups varied greatly. The intestinal flora of the DC group was disordered, and there were large differences from all other groups. The DC group was located on the right side of Axis 1, far from the normal group (NC). After treatment with tea polysaccharide and metformin, each group tended to be close to the NC group, and the different doses of the tea polysaccharide intervention group showed significant differences, indicating that tea polysaccharide and metformin had significant effects on the intestinal flora structure of rats. The MT group was the closest to the NC group, indicating that the effect of tea polysaccharide on T2DM rats was dose-dependent (Figure 9).

##### Non-Measured Multidimensional Scaling (NMDS) Analysis

Non-measured multidimensional scaling (NMDS) analysis differs from PCoA analysis by rank-ordering the sample distances so that the ranking of samples in the low-dimensional space conforms as closely as possible to the proximity of similar distances to each other (rather than to the exact distance values).

The smaller the stress value of the NMDS results, the better. The results of the NMDS analysis were generally considered more reliable when the value was less than 0.2. This modeling, STRESS = 0.0351, showed an excellent fit. Each point in the figure represents a sample, and the distance between the points indicates the similarity of their groups; the closer the distance is, the more similar the composition of the groups and the lower the beta diversity.

**Figure 10:**
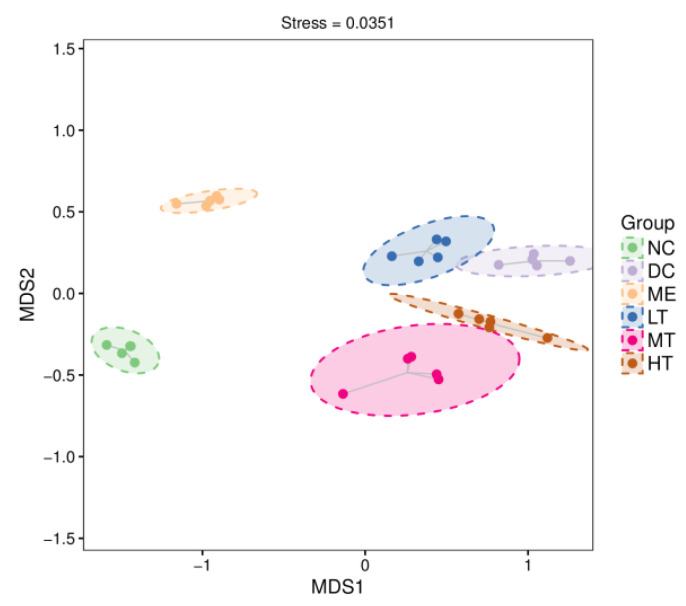
NMDS two-dimensional sorting diagram. Note: Each dot in the figure represents a sample, and dots of different colors indicate different treatment groups. Dashed oval circles are 95% confidence ellipses.

As shown in Figure 10, all sample groups could be separated well without crossover, showing a great difference between groups. Moreover, each tea polysaccharide intervention group was closer to the DC group than to the ME and NC groups, indicating that the flora of the LT, MT, and HT groups was closer to that of the DC group. The relative position of each tea polysaccharide intervention group also indicated a greater change in the flora composition of the MT group.

#### 3.3.4. Species Difference Analysis and Marker Species

##### Species Abundance Heatmap

Having explored the structural differences in the rat intestinal flora (beta diversity), we also need to know which differential distribution of the species was primarily responsible for these differences.

As seen from the heatmap, NC and ME, and LT and MT were on the same branch, indicating that the differences between these groups were smaller and that the composition of the flora was more similar. The differences between the DC and the NC and ME group could be seen to be larger; the differences between the DC and the LT and the MT and HT groups were seen to be smaller, consistent with the results of NMDS analysis and PCoA analysis.

**Figure A4:**
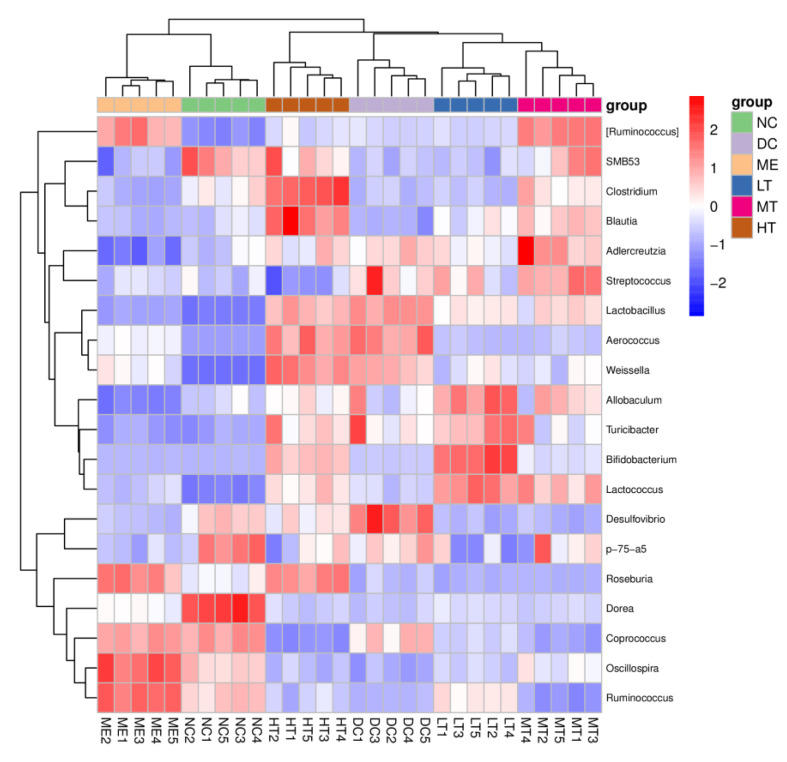
Heatmap of species composition at the genus level.

Compared to the NC group, *Adlercreutzia*, *Streptococcus*, *Lactobacillus*, *Aerococcus*, *Weissella*, *Turicibacter*, and *Desulfovibrio* were more abundant in the DC group; *SMB53*, *Lactococcus*, *Roseburia*, *Dorea*, *Coprococcus*, *Oscillospira*, *Ruminococcus*, and *Blautia* were less abundant in the DC group, suggesting that the structural composition of rat intestinal flora was altered after intraperitoneal injection of STZ. In contrast, after tea polysaccharide intervention, the abundance of the genera (*Ruminococcus*), *SMB53*, *Clostridium*, *Blautia*, *Allobaculum*, *Bifidobacterium*, *Lactococcus*, and *Ruminococcus* increased in the tea polysaccharide intervention groups relative to the DC group, while *Aerococcus*, *Desulfovibrio*, *p-75-a5*, and *Coprococcus* decreased in abundance. Moreover, *Clostridium* and *Blautia*, *Bifidobacterium* and *Lactococcus*, and *Desulfovibrio* and *p-75-a5* were on the same branch, indicating their strong correlation (Figure A4).

##### LEfSe Analysis

LEfSe emphasizes the search for biomarker species among subgroups, which has been widely used in the fields of microbial amplicon analysis and macrogenome analysis, and is especially suitable for finding biomarkers in medical research.

**Figure A5:**
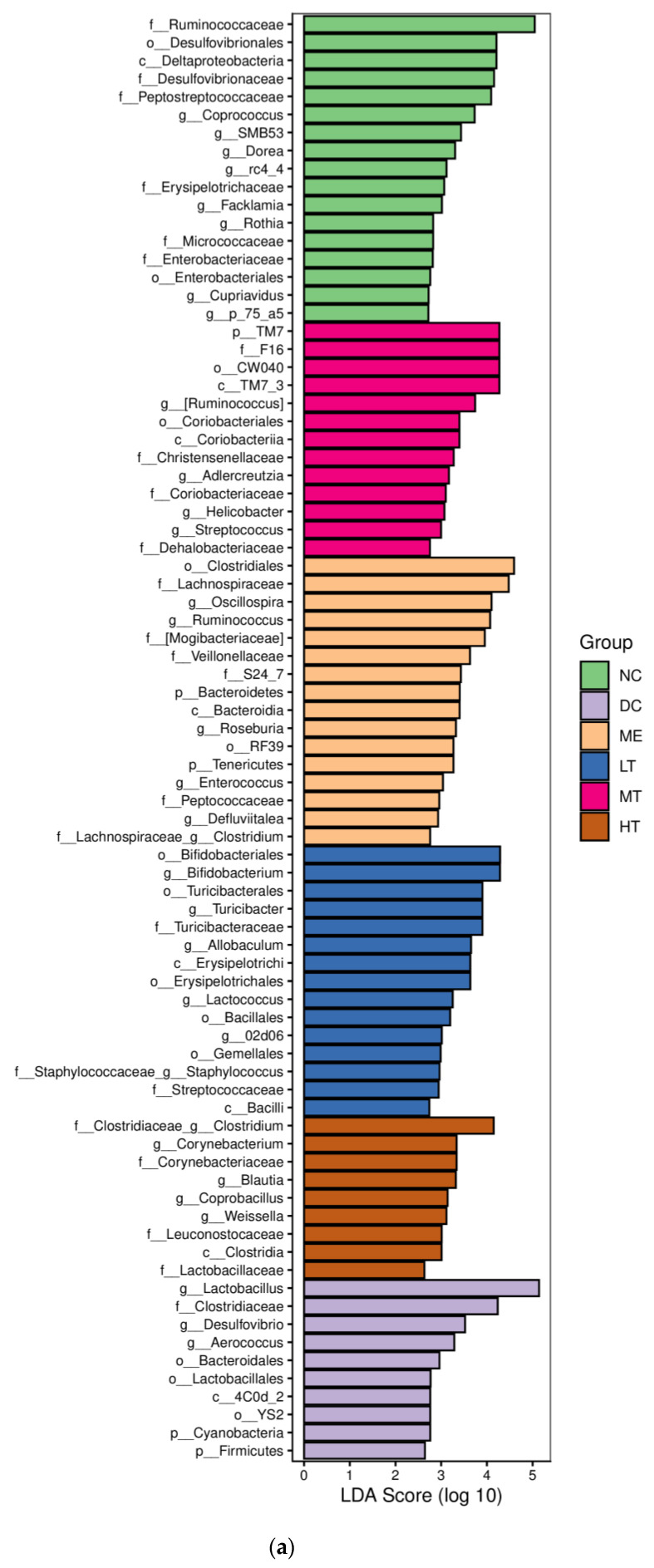

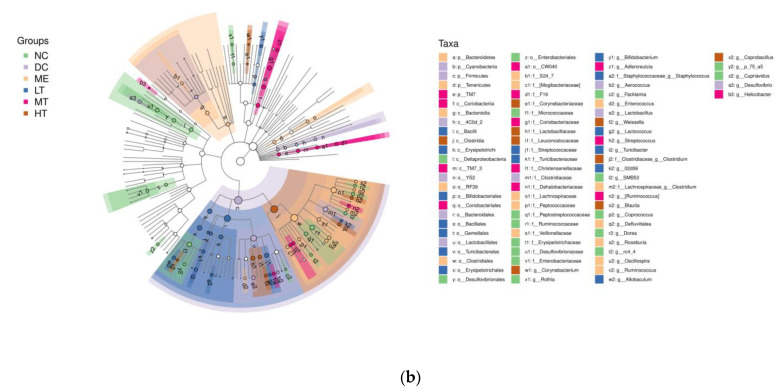
Linear discriminant analysis effect size (LEfSe) analysis of rat intestinal flora. (**a**) Histogram of LDA effect values for marker species; (**b**) display diagram of intergroup difference classification units based on classification hierarchy tree.

When LEfSe multilevel species difference analysis was applied to analyze the species differences of intestinal flora in each group (Figure A5), the abundance of *Lactobacillus*, *Desulfovibrio*, and *Aerococcus* in the intestinal flora of rats in the DC group was found to be significantly increased when LDA = 2. The intervention groups of rats with high, middle, and low doses of TPS for 40 days significantly improved the intestinal flora composition;the HT group significantly increased the intestinal flora of *Corynebacterium*, *Blautia*, *Coprobacillus*, *Weissella*; and the MT group significantly increased the abundance of (*Ruminococcus*), *Adlercreutzia*, *Helicobacter*, and *Streptococcus*. The LT group significantly increased *Bifidobacterium*, *Turicibacter*, *Allobaculum*, *Lactococcus*, and *02d06*. Among these genera whose growth was stimulated by tea polysaccharides, *Blautia*, *Weissella,* (*Ruminococcus*), *Bifidobacterium*, *Turicibacter*, *Lactococcus*, etc., have been shown to promote the secretion SCFAs and resist intestinal inflammation in previous reports [43].

These results suggested that TPS intervention in rats effectively increased the relative abundance of intestinal micro-organisms, which contributed to reducing the inflammatory response, increasing the production of SCFAs, and reducing the relative abundance of LPS-producing bacteria, such as *Desulfovibrio*. *Desulfovibrio* can induce a decrease in the level of SCFAs [44], which is associated with inflammatory bowel disease and T2DM [45].

The increased abundance of *Lactobacillus* in the DC group was quite surprising, as *Lactobacillus* was generally considered to be a healthy probiotic, meaning that the impact of intestinal flora on health was complex. Similarly, in an experimental study on pu-erh tea intervention for hypercholesterolemia, the relative abundance of *Lactobacillus* was found to be reduced in rat and human fecal samples at the genus level after pu-erh tea intervention [46]. Interestingly, pu-erh tea is a dark tea, and the tea polysaccharide content of dark tea is the highest among the six major types of tea. Different species of *Lactobacillus* may have different roles, which may explain why *Lactobacillus* is associated with health and microbial community diversity but is increased in model rats or diseased populations. Therefore, we may need to revisit the role and potential of *Lactobacillus* in preventing T2DM.

##### Random Forest Analysis

Random forest is a classical and efficient machine learning algorithm based on decision trees. This algorithm has been proven to be effective, robust, and accurate for classifying microbial community samples in recent years [31].

**Figure A6:**
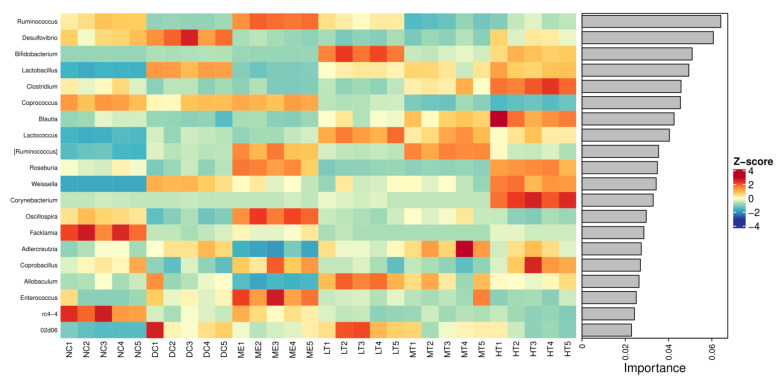
The heatmap of the top 20 most important genera.

The top 20 taxonomically important bacterial genera are shown in Figure A6, with species decreasing in importance from top to bottom in terms of influencing the grouping, and these top species in importance were considered to constitute the marker species for differences between groups. As shown in the figure, *Desulfoviobrio*, *Lactobacillus*, *Weissella*, and *02d06* were enriched in the DC group relative to the NC group, while *Ruminococcus*, *Clostridium*, *Facklamia*, and *rc4-4* decreased in abundance. Compared with the DC group, the ME group was enriched in *Ruminococcus*, (*Ruminococcus*), *Roseburia*, *Oscillospira*, *Coprobacillus*, and *Enterococcus* and decreased in *Desulfoviobrio*, *Lactobacillus*, *Weissella*, and *Adlercreutzia*. The abundances of *Ruminococcus*, *Bifidobacterium*, *Lactococcus*, and *Allobaculum* were enriched, while *Desulfoviobrio* and *Weissella* were decreased in the LT group. The abundances of *Clostridium*, *Blautia*, *Lactococcus*, and (*Ruminococcus*) were enriched; *Desulfoviobrio*, *Coprococcus*, and *Weissella* were decreased in the MT group. In the HT group, compared to the DC group, *Bifidobacterium*, *Clostridium*, *Blautia*, *Lactococcus*, *Roseburia*, and *Corynebacterium* were enriched; *Desulfoviobrio*, *Coprococcus*, and *02d06* were decreased in abundance.

*Desulfoviobrio*, enriched in the DC group, was considered a potentially pathogenic bacterium in previous studies; *Weissella* and *Lactobacillus* belong to lactic acid bacteria; and *Lactobacillus*, although usually considered a probiotic, was shown to be enriched in the intestine of the patient in several study reports [46,47]. It is possible that different species of *Lactobacillus* have different effects. The changes in the structure of the rat intestinal flora after interventions with different doses of tea polysaccharide varied, but the common denominator was the enrichment of *Ruminococcus*, *Bifidobacterium*, *Blautia*, and *Roseburia*, which are generally considered healthy bacteria and have been reported in the literature for their health-promoting effects. All three groups showed a decrease in the abundance of *Desulfoviobrio*, which were potentially pathogenic bacteria [45], indicating that tea polysaccharides can promote the enrichment of healthy bacteria and inhibit pathogenic bacteria.

##### Topological Indices and Hub Species

By analyzing the network topology, the relationship between important modules and possible key species was studied. Two main parameters were involved here: first, intramodule connectivity Zi (within-module connectivity), which was used to measure the degree of connectivity between nodes and other nodes in the module; second, intermodule connectivity Pi (among-module connectivity), which was used to quantify the degree of connectivity between nodes and other modules. According to Zi = 2.5 and Pi = 0.62, the nodes were divided into four major categories: (1) peripherals, with low values of Zi and Pi, which had few connections and were always connected to nodes inside the module; (2) connectors, with low Zi and high Pi, which had low connectivity inside the module but were highly connected to several modules; (3) module hubs, high Zi and low Pi, with many nodes in this module highly connected, but with few connections to other modules; and (4) network hubs, where Zi and Pi values were high, with both the role of module hubs and connectors.

**Figure 11:**
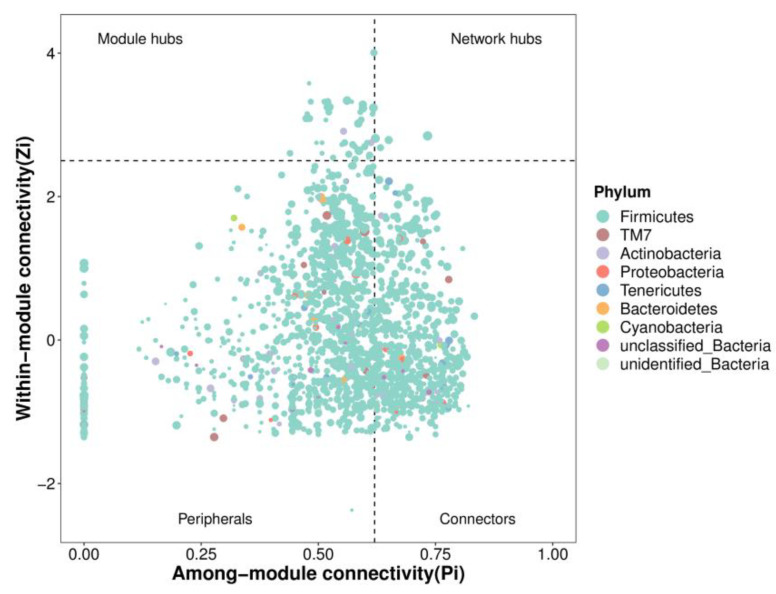
Zi-Pi scatter plot.

The topological roles of the network nodes in Figure 11 show that most of the network nodes were peripheral nodes, and that some of the peripheral nodes were not connected to outside modules (Pi = 0). The second most numerous nodes were connectors capable of showing a high degree of connectivity with multiple other modules. Module hub and network hub nodes with high values of both Zi and Pi had a high degree of connectivity with other modules, and were key species in this microecological community. The module hubs had a low number of nodes containing micro-organisms from two phyla, Firmicutes and Actinobacteria, while the network hubs had only a few nodes of Firmicutes. The abundances mentioned above vary greatly, such as *Ruminococcus*, *Lactobacillus*, *Blautia*, *Lactococcus*, and *Roseburia* belonging to Firmicutes. The presence of Actinobacteria in module hubs suggests that the low abundance of micro-organisms may also be among the key groups that play an important role in the intestinal flora network. *Bifidobacterium*, which was significantly enriched by tea polysaccharide intervention, belongs to Actinobacteria.

##### Analysis of Metabolic Pathway Differences

**Figure A7:**
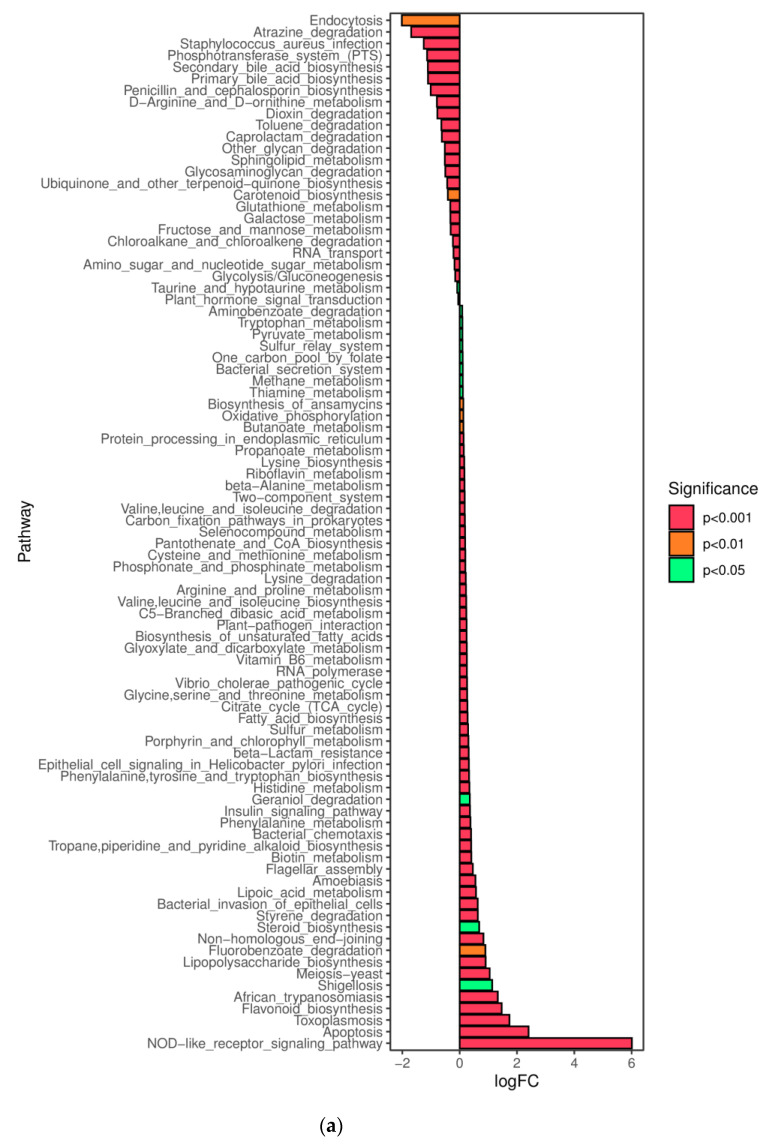

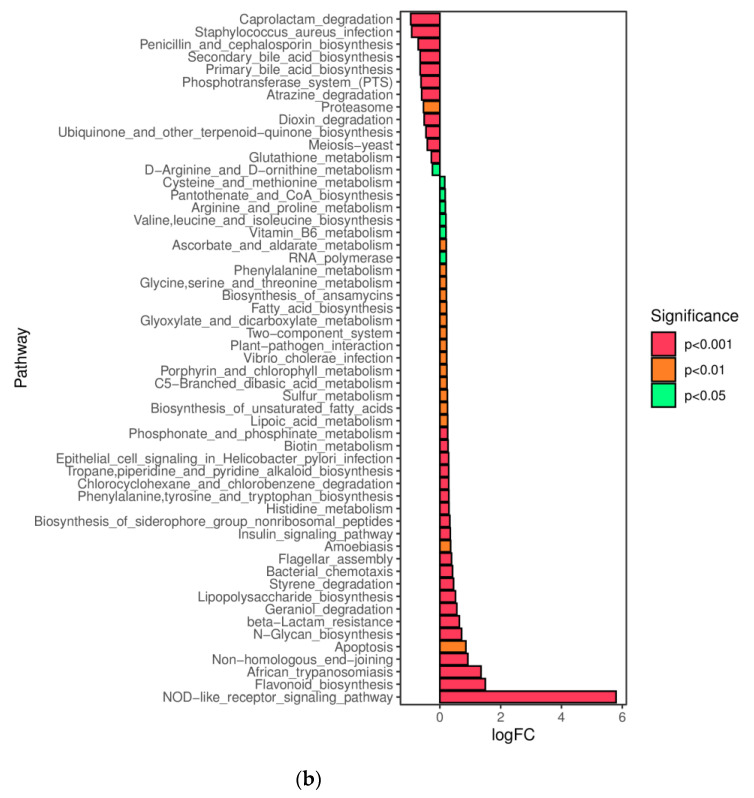

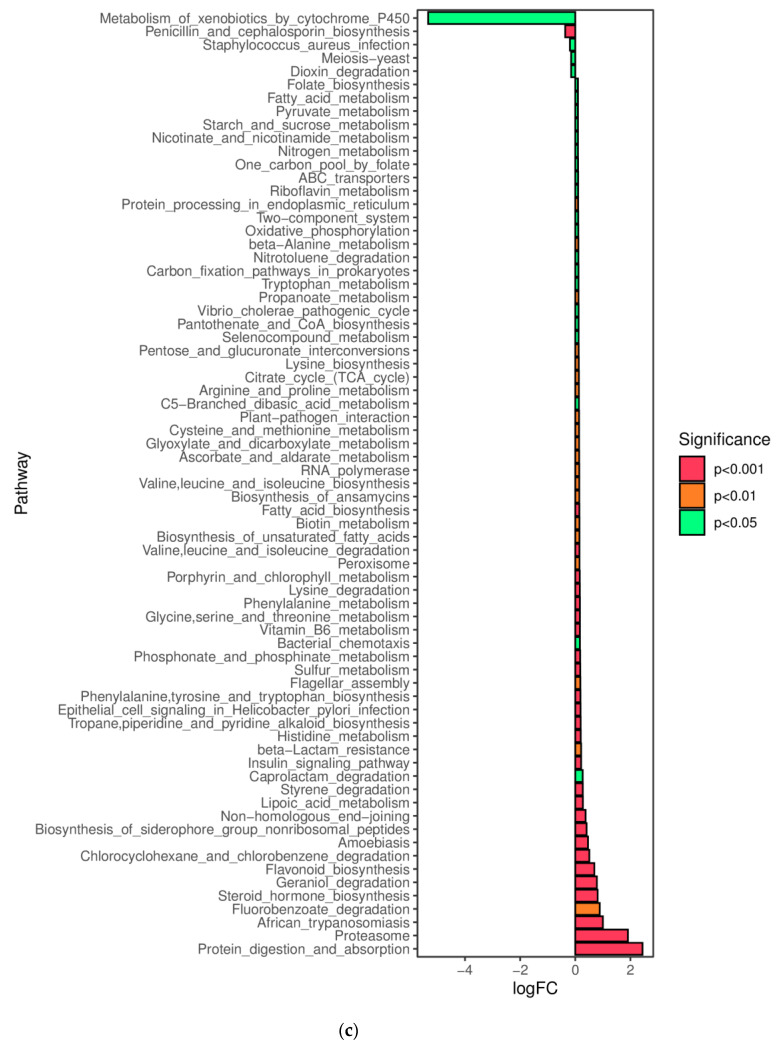

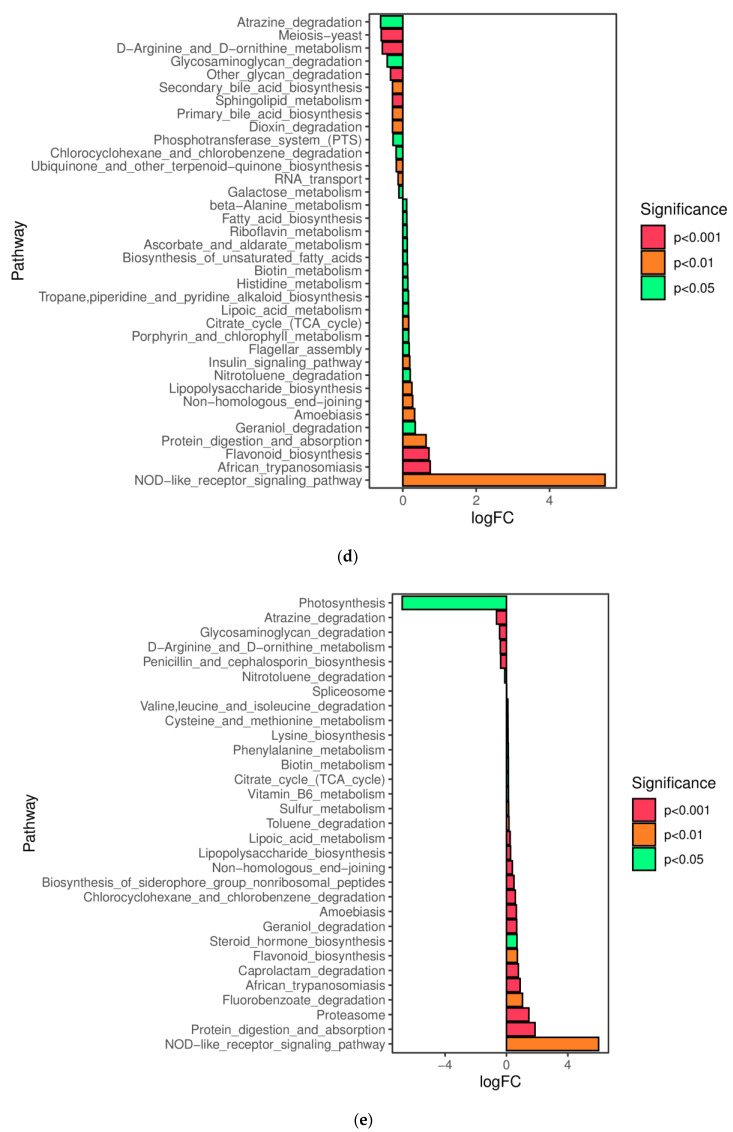
Intergroup differences in the KEGG metabolic pathway. (**a**) DC vs. NC; (**b**) DC vs. ME; (**c**) DC vs. LT; (**d**) DC vs. MT; (**e**) DC vs. HT. Note: Positive values of logFC on the horizontal axis represent upregulation (log2(fold change)) in DC group relative to group of NC, ME, LT, MT, and HT, respectively, and negative values are downregulation; vertical coordinates are different KEGG metabolic pathway labels; the degree of significance is shown in different colors.

PICRUSt2 (Phylogenetic Investigation of Communities by Reconstruction of Unobserved States) was used to predict the functional abundance of the samples. After counting the number of abundances of secondary functional pathways in the KEGG database, metagenomeSeq was used to identify metabolic pathways with significant differences between groups (DC vs. NC, DC vs. ME, DC vs. LT, DC vs. MT, DC vs. HT)(Figure A7).

The results showed that 64 metabolic pathways, such as the NOD-like receptor signaling pathway, apoptosis, lipopolysaccharide biosynthesis, and insulin signaling pathway, were significantly improved; 23 metabolic pathways, such as endocytosis, secondary bile acid biosynthesis, and primary bile acid biosynthesis, were significantly decreased in the DC group compared with the NC group (*p* < 0.05). The comparison of the metabolic pathways in the DC group with those of tea polysaccharide intervention groups (HT, MT, and LT) showed that the NOD-like receptor signaling pathway, insulin signaling pathway, and lipopolysaccharide biosynthesis were more likely to be upregulated in the DC group, while secondary bile acid biosynthesis and primary bile acid biosynthesis were more likely to be downregulated.

## 4. Discussion

The structure of polysaccharides determined their biological activities. Numerous studies have shown that the differences in the biological activity of tea polysaccharides obtained from different tea raw materials or different preparation methods were due to structural differences in tea polysaccharides, such as molecular weight, monosaccharide composition, glycosidic bond linkage form, conformation, etc. [7,18,48,49].

Chen et al. summarized the previous tea polysaccharides extracted from different tea materials, and their molecular weights were distributed mainly between 1.2 KD and 3900 KD [50]. Lu et al. reported that TPS in Huangshan Mao Feng green tea was an acidic heteropolysaccharide, and that the monosaccharides contained mannose, ribose, rhamnose, gluconic acid, galacturonic acid, glucose, xylose, galactose, and arabinose, as determined by high-performance liquid chromatography (HPLC) [18]. In this study, we found that the TPS from yellow leaves of Wuyi rock tea was mainly composed of rhamnose, arabinose, galactose, glucose, and galacturonic acid.

The biological activity of polysaccharides was closely related to their structure and conformation. Structure–activity relationship analysis revealed that the biological activity of yam polysaccharides might be more dependent on their higher molecular weight, higher galacturonic acid content, and complex spatial configuration [51]. Ren et al. found that polysaccharides of appropriate molecular weight could ensure their easy entry into cells through tissue barriers, which greatly improved their biological efficiency in inhibiting viral replication [52]. Surenjav et al. confirmed that the biological activity of polysaccharides required polysaccharides with a regular advanced structure [53].

As a biological macromolecule, tea polysaccharide was relatively difficult to be absorbed by cells. As a polysaccharide containing a galacturonic acid structure, tea polysaccharide was less efficiently absorbed by the small intestinal mucosa due to the electrostatic repulsive force between the negatively charged tea polysaccharide containing a carboxylic acid group and the negatively charged small intestinal mucosa, which reduced its bioavailability [11].

In contrast, micro-organisms colonizing the human gut had broader genetic heterogeneity and greater degrading enzymes and metabolic capabilities than their hosts. In general, Bacteroides have relatively large genomes that encode various carbohydrate-active enzymes (CAZymes) that confer the ability to synthesize, recognize, or metabolize complex carbohydrates in bacteria. Zeng et al., utilized polysaccharides from Fuzhuan brick tea as a material, and the relative molecular mass, polysaccharide content, and reducing sugar content of TPS did not change after digestion by the digestive system (simulated saliva, gastric juice, and small intestine fluid), indicating that TPS could “escape” digestion of the digestive system and reach the large intestine safely. After fermentation by simulated intestinal flora in vitro, the polysaccharide content was significantly reduced, indicating that TPS could be degraded and utilized by intestinal micro-organisms, implying that the physiological activity of tea polysaccharides might act through the intestinal flora [54]. Moreover, the intestinal flora is considered to play a crucial role in promoting host health, and is therefore often referred to as the “forgotten organ”. Studies have shown that intestinal microbial imbalance might be a key environmental factor for various complex diseases. With the rapid development of high-throughput sequencing technology, the relationship between human intestinal flora and T2DM is gradually being revealed [55].

In our study, *Lactobacillus* was significantly increased in the intestines of type 2 diabetic rats. *Bifidobacterium* rebounded significantly with the intervention of tea polysaccharides. *Lactobacillus* and *Bifidobacterium* can train the immune system, inhibit the reproduction of other harmful bacteria, are often considered probiotics, and have been validated in many studies. However, there were also some contradictory reports. Professor Zhao and colleagues intervened in patients fed a high dietary fiber diet and found that the selectively enriched intestinal flora could alleviate type 2 diabetes, among which *Bifidobacterium pseudocatenulatum* was one of the most significant strains to promote SCFA production [55]. Stephanie Schnorr and colleagues investigated the phylogenetic diversity and metabolite production of the gut microbiota from a community of human hunter–gatherers, the Hadza of Tanzania. They showed that the gut of Hadza had an absence of *Bifidobacterium*, suggesting that *Bifidobacterium* was not necessary for survival [56]. Mohamed Elfil and his colleagues [47] reported a significant increase in *Lactobacillus* in peritoneal dialysis (PD) patients. In an experimental study on pu-erh tea intervention for hypercholesterolemia, the relative abundance of OTUs of *Lactobacillus*, *Bacillus*, *Lactococcus*, and *Streptococcus* was found to be reduced in rat and human fecal samples at the genus level after pu-erh tea intervention. The author believed that the common function of these micro-organisms with reduced abundance was the production of BSH enzymes [46]. Chen and colleagues used five-week-old male mice fed a high-energy diet with different lipid-to-sugar ratios to induce prediabetic mice. The intestinal flora is characterized by a high-fat diet rich in *Lactobacillus* and *Bifidobacterium* [57]. Taken together with previous studies, the intestinal flora of individuals with significant T2DM and prediabetes showed a relative decrease in butyrate-producing bacteria and an increase in species with potential proinflammatory functions [58]. However, attempts to transfer intestinal flora from unmedicated individuals with T2DM disease or prediabetes to germ-free mice to replicate the T2DM phenotype have thus far been unsuccessful [58], thus also illustrating the complexity of the relationship between intestinal flora and T2DM.

In short, because of the wide variation among individuals of different populations, races, genders, ages, and health conditions, there may be no “gold standard” for human gut flora that promotes metabolically healthy hosts. Associating gut microbiota and host disease with a single mechanism is not desirable.

## 5. Conclusions

In this study, a novel polysaccharide from yellow leaves of Wuyi rock tea named CY was isolated and purified. The mean MW value of CY was 39.285 kDa. CY was mainly composed of rhamnose, arabinose, galactose, glucose, and galacturonic acid, the majority of which mainly contained 10 kinds of glycosyl residues, α-l-Araf-(1→, →5)-α-l-Araf-(1→, →4)-α-d-Glcp-(1→, Arap-(1→, →6)-α-d-Glcp-(1→, →2,4)-α-l-Rhap-(1→, →3,4)-α-d-Glcp-(1→, →4)-α-d-GalAp-(1→, α-d-Galp-(1→, →6)-β-d-Galp-(1→, →4)-β-d-Galp-(1→. CY intervention significantly lowered the blood sugar levels in type 2 diabetic rats. Both α-diversity and β-diversity indices provided strong evidence for microbial structural dysbiosis in T2DM rats. Gut microbiota analysis revealed that Actinobacteria play an important role in the intestinal flora network. *Bifidobacterium* was the key micro-organism that promoted significant improvements in flora structure. Moreover, the metabolic functions of the gut microbiota were enhanced based on bioinformatics analysis. Gene expressions including secondary bile acid biosynthesis and primary bile acid biosynthesis were upregulated in the metabolic pathway. Gene expressions including the NOD-like receptor signaling pathway, lipopolysaccharide biosynthesis, and insulin signaling pathway were downregulated in the metabolic pathway. Hence, CY might be used to treat glucose metabolism disorders and their complications as a food supplement.

## Figures and Tables

**Table 1:** HPGPC analysis of CY.

Sample ID	RT(min)	lgMp	lgMw	lgMn	Mp	Mw	Mn
	39.76	4.5	4.6	4.5	32,729	39,285	28,224

## Data Availability

The data presented in this study are available on request from the corresponding author, upon reasonable request.

## Appendix A

**Table A1:** Monosaccharide Composition Analysis of CY.

Name	RT	Linear	R2	Range (μg/mL)	Molar Ratio	Quality Ratio (%)
Fucose (Fuc)	5.875	y = 0.184x + 0.98	0.990	1–100	0.000	0.000
Rhamnose (Rha)	11.75	y = 0.089x + 0.226	0.996	1–100	0.086	7.680
Arabinose (Ara)	12.742	y = 0.243x + 1.096	0.990	1–100	0.124	10.127
Glucosamine hydrochloride (GlcN)	14.067	y = 0.427x + 1.963	0.992	1–100	0.000	0.000
Galactose (Gal)	16.275	y = 0.242x + 1.161	0.997	1–100	0.143	14.014
Glucose (Glc)	18.417	y = 0.259x + 1.715	0.990	1–100	0.020	1.961
N-acetyl-d-glucosamine (GlcNAc)	19.717	y = 0.195x + 0.549	0.997	1–100	0.000	0.000
Xylose (Xyl)	21.55	y = 0.152x + 0.815	0.993	1–100	0.000	0.000
Mannose (Man)	22.384	y = 0.095x + 0.331	0.996	1–100	0.000	0.000
Fructose (Fru)	25.809	y = 0.075x − 0.148	0.997	1–100	0.000	0.000
Ribose (Rib)	27.859	y = 0.62x − 0.494	0.996	1–100	0.000	0.000
Galacturonic acid (GalA)	45.542	y = 0.155x + 0.004	1.000	1–100	0.627	66.218
Glucuronic acid (GlcA)	49.075	y = 0.192x + 0.195	0.999	1–100	0.000	0.000

**Table A2:** The methylation of CY.

Retention Time (min)	Methylated Sugar	Mass Fragments (*m*/*z*)	Molar Ratio	Type of Linkage
9.45	2,3,5-Me_3_-Araf	43, 71, 87, 101, 117, 129, 145, 161	2.54	Araf-(1→
10.856	2,3,4-Me_3_-Arap	43, 71, 87, 101, 117, 129, 131, 161	8.31	Arap-(1→
14.435	2,3-Me_2_-Araf	43, 71, 87, 99, 101, 117, 129, 161, 189	3.10	→5)-Araf-(1→
17.425	2,3,4,6-Me_4_-Galp	43, 71, 87, 101, 117, 129, 145, 161, 205	8.49	Galp-(1→
18.515	3-Me_1_-Rhap	43, 87, 101, 117, 129, 143, 159, 189	8.37	→2,4)-Rhap-(1→
18.747	2,3,6-Me_3_-Galp	43, 87, 99, 101, 113, 117, 129, 131, 161, 173, 233	42.15	→4)-Galp-(1→
21.107	2,3,6-Me_3_-Glcp	43, 87, 99, 101, 113, 117, 129, 131, 161, 173, 233	5.40	→4)-Glcp-(1→
21.345	2,3,4-Me_3_-Galp	43, 87, 99, 101, 117, 129, 161, 189, 233	9.98	→6)-Galp-(1→
24.278	2,6-Me_2_-Glcp	43, 87, 97, 117, 159, 185	3.06	→3,4)-Glcp-(1→
27.762	2,3-Me_2_-Glcp	43, 71, 85, 87, 99, 101, 117, 127, 159, 161, 201	8.60	→4,6)-Glcp-(1→

**Table A3:** Assignment of ^13^C and ^1^H chemical shifts of CY.

Glycosyl Residues	H1	H2	H3	H4	H5a/H5	H5b/6a	H6b	CH_3_	CH_3_CO
	C1	C2	C3	C4	C5	C6			
α-L-Araf-(1→	5.17	4.13	3.87	4.06	3.76	3.64			
	110.62	82.62	77.97	85.22	62.64				
→5)-α-L-Araf-(1→	5.04	4.07	3.96	4.16	3.84	3.75			
	108.6	82.52	78.12	83.6	68.09				
→4)-α-D-Glcp-(1→	5.31	3.5	3.86	3.55	3.76	3.73	3.76		
	100.88	72.75	74.53	77.98	72.45	61.73			
Arap-(1→	5.09								
	108.76								
→6)-α-D-Glcp-(1→	4.89	3.48	3.63	3.44	3.83	3.9	3.66		
	99.14	72.84	74.7	70.98	71.44	66.79			
→2,4)- α-L-Rhap-(1→	5.2	4.04	4.02	4.33	3.8	18			
	99.72	77.57	71.63	78.47	70.87	1.17			
→3,4)-α-D-Glcp-(1→	4.95	3.8	4.03	4.36	4.7	18			
	99.14	70.75	77.48	80.11	72.97	1.17			
→4)-α-D-GalAp-(1→	4.88	3.66	3.91	4.36	5.05			3.73	1.96
	101.7	69.54	69.79	80.06	72.09	172.13		54.29	21.2
→4)-α-D-GalAp-(1→	5.04	3.73	3.94	4.52	4.6				
	101.87	69.8	70.1	80.78	72.92	176.24			
α-D-Galp-(1→	5.04	3.73	3.94	4.03	4.6	3.66	3.75		
	101.87	69.8	70.1	71.74	72.92	62.77			
→6)-β-D-Galp-(1→	4.37	3.44	3.58	3.86	3.88	3.61	3.83		
	104.9	72.16	73.93	74.96	69.87	76.1			
→4)-β-D-Galp-(1→	4.56	3.6	3.7	4.08	3.65	3.74			
	105.74	73.12	74.61	79.05	75.81	62.11			

**Table A4:** The effects of CY on the blood glucose of hyperglycemic rats ($\overset{-}{x}$ ± s).

Group	Blood Glucose Concentration/(mmol/L)				
	0 d	10 d	20 d	30 d	40 d
NC	5.14 ± 0.16	4.78 ± 0.49	5.28 ± 0.53	4.82 ± 0.76	5.19 ± 1.11
DC	24.45 ± 3.87 ^ΔΔ^	25.27 ± 3.61 ^ΔΔ^	23.56 ± 5.02 ^ΔΔ^	24.32 ± 5.27 ^ΔΔ^	24.17 ± 3.13 ^ΔΔ^
ME	27.47 ± 3.56	26.36 ± 4.68	22.05 ± 2.67	19.16 ± 3.85	16.01 ± 2.78 *
LT	25.74 ± 2.13	23.17 ± 4.93	21.22 ± 4.02	19.37 ± 4.45	19.42 ± 3.38
MT	27.30 ± 2.02	22.73 ± 5.17	20.81 ± 4.29	19.87 ± 4.78	17.12 ± 2.15 *
HT	28.65 ± 4.78	27.54 ± 3.94	23.56 ± 4.05	18.97 ± 3.15	19.25 ± 3.02

Note: (1) 0 d shows the blood glucose values before treatment; 10 d, 20 d, 30 d, and 40 d show the blood glucose values at 10, 20, 30, and 40 days of drug administration, respectively; (2) ^ΔΔ^ represents highly significant difference (*p* < 0.01) between DC group and NC group control. * Represents a significant difference (*p* < 0.05) between the drug administration group and DC group.

**Table A5:** The effects of CY on glucose tolerance of hyperglycemic rats ($\overset{-}{x}$ ± s).

Groups	Blood Glucose Concentration/(mmol/L)				
	0 min	30 min	60 min	120 min	AUC
NC	4.49 ± 1.20	5.72 ± 0.93	5.31 ± 0.62	4.62 ± 0.54	7.79 ± 1.18
DC	22.86 ± 2.74 ^ΔΔ^	31.27 ± 2.65 ^ΔΔ^	23.68 ± 2.77 ^ΔΔ^	23.22 ± 3.26 ^ΔΔ^	39.00 ± 3.52 ^ΔΔ^
ME	15.78 ± 1.88 *	22.62 ± 1.82 *	20.59 ± 2.16	16.20 ± 2.00 *	29.60 ± 2.26 **
LT	18.85 ± 2.69	25.53 ± 2.72	23.64 ± 3.28	19.92 ± 2.56	34.27 ± 3.05 *
MT	17.53 ± 1.84	24.17 ± 2.21 *	22.18 ± 2.09	18.67 ± 1.94	32.23 ± 2.74 **
HT	17.68 ± 2.26	25.58 ± 3.14	21.91 ± 2.55	19.32 ± 2.91	32.99 ± 3.32 **

Note: (1) 0 min shows the blood glucose values before treatment; 30 min, 60 min, and 120 min show the blood glucose values at 30, 60, and 120 min of drug administration, respectively; (2) ^ΔΔ^ represents highly significant difference (*p* < 0.01) between DC group and NC group control. * Represents a significant difference (*p* < 0.05); ** represents a highly significant difference (*p* < 0.01) between the drug administration group and DC group.

**Table A6:** The effects of CY on biochemical indices in diabetic rats.

Group	Biochemical Indices					
	TG	TC	LDL-C	HDL-C	AST	ALT
NC	0.57 ± 0.15	2.17 ± 0.13	0.55 ± 0.07	1.58 ± 0.16	142.80 ± 8.96	93.24 ± 9.57
DC	3.99 ± 0.28 ^ΔΔ^	3.56 ± 0.35	1.36 ± 0.11 ^ΔΔ^	1.08 ± 0.17 ^Δ^	198.00 ± 31.27	107.05 ± 13.74
ME	2.19 ± 0.06 **	2.74 ± 0.16	0.77 ± 0.08 **	1.17 ± 0.20	123.54 ± 11.52	79.61 ± 9.63
LT	2.91 ± 0.25 *	2.54 ± 0.47	1.27 ± 0.13	1.29 ± 0.25	128.43 ± 9.12	87.94 ± 5.46
MT	2.67 ± 0.17 **	2.55 ± 0.38	1.25 ± 0.02	1.29 ± 0.11	117.73 ± 6.82 *	86.90 ± 5.75
HT	2.85 ± 0.13 *	2.61 ± 0.32	0.87 ± 0.12 *	1.23 ± 0.19	127.30 ± 14.06	75.36 ± 5.26

Note: ^Δ^ represents a significant difference (*p* < 0.05); ^ΔΔ^ represents a highly significant difference (*p* < 0.01) between the DC group and NC group control; * represents a significant difference (*p* < 0.05) between the drug administration group and DC group; ** represents a highly significant difference (*p* < 0.01).
